# The Use of Anagrelide in Myeloproliferative Neoplasms, with Focus on Essential Thrombocythemia

**DOI:** 10.1007/s11899-016-0335-0

**Published:** 2016-08-06

**Authors:** Gunnar Birgegård

**Affiliations:** Department of Medical Sciences, Uppsala University , Entrance 40, Universtiy Hospital, Uppsala, 75185 Sweden

**Keywords:** Anagrelide, Essential thrombocythemia, ET, Platelets

## Abstract

Anagrelide (ANA) is a drug with specific platelet-lowering activity, used primarily in ET, registered as a second-line drug in essential thrombocythemia (ET) in Europe and in some countries as first-line therapy, in USA licensed by FDA for thrombocythemia in myeloproliferative neoplasms (MPN). The platelet-lowering efficacy is similar to that of hydroxycarbamide (HC), around 70 % complete response and 90 % partial response. Side effects are common, especially headache and tachycardia, but usually subside or disappear within a few weeks. Around 20 % of patients stop ANA therapy due to side effects or insufficient response. Studies of treatment patterns in Europe show that ANA is preferentially given to younger patients, probably because of the concern for a possible leukemogenic effect of the common first-line drug, HC. Only two randomized studies have compared the efficacy of ANA and HC in preventing thrombosis and haemorrhage, the larger of them showing a slightly better efficacy of HC, the other showing non-inferiority of ANA to HC. A recent observational 5-year study of 3600 patients shows a low and basically similar efficacy of ANA and other cytoreductive therapies in ET. ANA does not appear to inhibit fibrosis development, and probably due to its anticoagulation properties, the combination of ASA and ANA produces an increased rate of haemorrhage. Combination of ANA with HC or interferon (IFN) is feasible and effective in patients with insufficient platelet response to mono-therapy.

## Introduction, Background

The goal of treatment in ET is reduction of thrombotic and haemorrhagic complications, and the target is platelet reduction to <400 × 10(9)/L [1]. There is no drug that can prolong life in these patients, and with the almost normal survival in true ET, any survival effect of a new drug will be very difficult to prove. ET patients have more general symptoms than previously believed [2], and it is unclear how platelet reduction or general cytoreduction relieves symptoms[3]. In extreme thrombocythemia, there is an increased risk of haemorrhage, partly due to a secondary Von Willebrand disease [4, 5], and one large study found the risk of haemorrhage to be increased already in platelet levels above normal during maintenance treatment [6]. Aspirin treatment increases bleeding risk in patients with high platelet levels [7], and the platelet level should therefore be lowered by cytoreduction in patients with platelets above 1000 × 10(9)/L before starting aspirin therapy [8].

There is only one study of thrombosis rate in ET with a control arm without treatment [9]. Although this study had a limited number of participating patients (*n* = 114) and during a follow-up time of 27 months had a total of only 16 thrombotic events, the results were statistically significant, with 2 thrombotic events in the treatment arm (HC) vs 14 in the control arm (*p* = 0.003). For ethical reasons, a study with an untreated arm has since then not been considered possible. ET patients are classified as high or low risk based on age (>60 years), prior thrombohaemorrhagic event or platelets >1500 × 10(9)/L (bleeding risk), and all patients with any of these criteria are considered high risk patients eligible for cytoreductive treatment [1]. Recently, attempts have been made to include other possible risk factors in risk score models, based on studies showing correlations between thrombosis risk and presence of JAK2V617F mutation, cardiovascular risk factors or white blood cell (WBC) counts. Cardiovascular risk factors have consistently been found to increase thrombosis risk in ET, whereas in the other two JAK2 positivity showed significance in one model [10] but not in another [11], and WBC count in neither of these two. The most ambitious risk score project is the IPSET-thrombosis [10], which has been validated retrospectively but not prospectively, and contains age >60, thrombosis history, cardiovascular risk and JAK2 positivity. A later modification of the IPSET-thrombosis score suggests that an intermediate risk category should be used, containing low risk patients with JAK2 mutation and CV risk factors [12]. Presence or absence of the newly found calreticulin (CAL-R) mutation has been shown not to influence the IPSET model [13].

The goal of treatment in ET is reduction of thrombosis and haemorrhage, and the means to achieve this is platelet reduction, preferably to the normal range, in spite of the recent findings of a negative correlation between high platelets at diagnosis and previous thrombosis [14, 15]. Further, two recently published registry studies showed a correlation between higher platelet counts during maintenance treatment and thrombosis at the time of event [16, 17•].

### Mode of Action

ANA is an amidazoquinazolin, originally developed as an anticoagulation drug, which was shown to have a potent platelet reducing effect [18]. It is the only platelet-specific cytoreductive drug known, having no inhibitory effects on red or white cell progenitor proliferation [19]. It reduces platelet production by inhibiting megakaryocyte (MK) colony development, thus producing a left-shift in MK maturation, reducing MK size, ploidy and maturation. Contrary to hydroxycarbamide (HC), which has its action mainly in the early phases of MK production, lowering MK proliferation, the effect of ANA mostly affects differentiation [19–21]. A substantial increase in thrombopoietin (TPO) levels has been found [22]. The platelet-reducing effect is mediated through reduction in pro-platelet formation [23]. GATA-1, which is the founding member of the GATA family of growth factors, is essential for the maturation and differentiation of MKs. This factor is upregulated in bone marrow cells in ET, and ANA has been shown to repress *GATA-1* [24] and *FOG-*1 expression [25], indicating that ANA works on an upstream regulator of GATA-1 and FOG-1. Recently, gene expression studies have revealed that a large number of genes are differentially regulated by ANA. ANA induced the phosphorylation of eIF2a, which is an upstream regulator of ATF4, and increased ATF4 protein levels, suggesting new targets for anti-MK treatment [26••]. The anti-MK effect is not accompanied by dysplasia of the bone marrow [21] and is separate from the PDIII-reducing effect responsible for the most common side effects [23]. Even though many details of the mechanism of action of ANA have been elucidated, the overall picture is still not clear. There are two slightly different formulations of anagrelide with some pharmacological differences. Xagrid® (Shire Pharmaceuticals) is registered in Europe as an orphan drug for second-line therapy in ET. It is rapidly metabolized into 3-hydroxyanagrelide and a major metabolite, 2-amino-5,6-dichloro-3,4-dihydroquinazoline (RL 603). Cyclic adenosine monophosphatedependent PDE3A has been identified as the molecular target for the former, whereas the platelet reducing effect is mainly mediated through RL 603 [27]. Thromboreductin® (AOP Orphan Pharmaceuticals AG), which is registered in some countries, is anagrelide hydrochloride and has a slower uptake and a lower Cmax of anagrelide in plasma in a study in healthy volunteers [28], where also a lower rate of side effects was noted (see below). No difference has been found in the pharmacokinetics between elderly and younger patients [29].

## Platelet-Lowering Efficacy

In an early study from the American Anagrelide Study group [30] 577 patients, mostly ET (335) with thrombocythemia, a majority of whom were resistant to other therapy, were treated with a resulting ≥50 % reduction of platelets in 93 % of the patients. Even though these results have been questioned for lack of strict definitions, concomitant use of HC and short treatment time [31], they stimulated to further use of ANA. Smaller phase II-III studies [32–34] confirmed the platelet-lowering effect, but no larger clinical study was published until 1997, when >900 patients were reported (by further accrual to the 1992 study) with a complete response rate of 70 %.[35] Response was seen within a week, and target platelet level reached within 2 weeks. This differs from later studies, the likely reason being that higher initial doses of 1-g bid were used. This produced a high frequency of side effects, and later studies therefore used the starting dose of 0.5-mg bid, which became standard. Since response criteria and cohort definitions vary between studies, a meta-analysis of response rates is difficult to perform, but including studies with >30 patients, a survey found a complete platelet response rate of ∼70 % in ET, complete plus partial response rate >90 %, somewhat lower in PV [31]. The platelet-lowering effect with recommended dose is similar to standard doses of HC but faster than for interferon (IFN): a treatment goal of platelets <400 × 10(9)/L was reached within 8–10 weeks [36] and 12–16 weeks [37, 38•]. In another study, a goal of <600 × 10(9)/L was reached within 8 weeks [39]. Studies reporting on more than 10 years experience with ANA treatment were published at the turn of the century [40, 41], showing that ANA was used in clinical practice quite early; however, no randomized study was published until 2005. Two small recent studies [42, 43] suggest that ANA may be more effective in JAK2-positive patients than in CALR-positive, but this needs further study.

## Clinical Efficacy (Thrombosis Prophylaxis)

### Thrombosis Reduction

The evidence for ANA in this respect rests partially on historical controls, showing a lower incidence of thrombotic events after start of ANA treatment [44]. Two randomized studies have been published, comparing ANA and HC efficacy. The first comprised 809 high-risk ET patients, diagnosed by PVSG criteria, followed for 39 months, randomized to ANA or HC, all receiving ASA [37], the PT1 study. Both treatments gave a low rate of thrombohemorrhagic events, around 2–3 %, comparable to the treatment arm in the Cortelazzo study (3.6 %), in contrast to the untreated arm in this study (24 %). In the PT1 study, HC patients had a significantly lower number of arterial thrombosis (17 vs 37, *p* = 0.004) and haemorrhage (8 vs 22, *p* = 0.008) but a higher number of venous thrombosis (14 vs 3, *p* = 0.006). The second study (38), the ANAHYDRET study, was a non-inferiority study with fewer patients (*n* = 259), all high-risk ET patients diagnosed according to the new WHO criteria of 2008 and followed for 36 months. This study found no significant difference in the rate of arterial or venous thrombosis or haemorrhage. A direct comparison between these studies is difficult, since the diagnostic criteria used were different, the size of the cohort studied was different and ASA was given to all patients in one study but not in the other. The ANAHYDRET study investigated a cohort defined by stricter criteria, a true ET population, but on the other hand maybe was too small to detect a difference in efficacy. The largest study to date, the EXELS study, is a non-randomized, prospective, observational study where >3600 high-risk ET patients on cytoreductive therapy were followed for 5 years. More than 80 % of the patients had either ANA or HC therapy, and due to the preferences of treating physicians the ANA cohort was younger (56 vs 70 years). The event rates of total thrombosis and arterial thrombosis were slightly lower in the ANA group, as may be expected from the age difference, whereas the venous thrombotic rate was considerably lower, consistent with the finding in the PT1 study. The event rate of thrombosis and haemorrhage was generally low for both treatments, around 2.5 % (Birgegård et al., abstract 1846, ASH 2014).

### Anagrelide and Bone Marrow Fibrosis

In the PT1 study, a higher frequency of transformation to myelofibrosis was shown (16 vs 5, *p* = 0.01) in the ANA group, and another study found progression of fibrosis in 19 ET patients after 2 years of ANA treatment [45], whereas the ANAHYDRET study found no fibrosis development. In the EXELS study, where ET diagnosis was made using both older and newer diagnostic criteria, the transformation event rate for MF was also higher in the ANA-treated patients. One may conclude from these results that ANA does not prevent fibrosis development, but it cannot be concluded that it provokes fibrosis development. It seems obvious that the optimal treatment group for ANA is true ET patients, since it has been shown that fibrosis development is a much rarer event in WHO-diagnosed true ET than in patients with older diagnostic criteria [46•, 47]. However, in MF patients with lack of response to HC anagrelide of course can be used as second-line therapy. In patients with reticulin fibrosis grad 2 or more, treated with ANA, progress of fibrosis by bone marrow biopsy should be considered, maybe every second year.

### Side Effects

Side effects are common during the first weeks of treatment, but usually subside [36, 37, 40]. The most common side effects, tachycardia, headache and dizziness are probably due to phosphodiasterase III-inhibiting properties of ANA [48] and specifically by one of its metabolites, 3-hydroxyanagrelide [23]. Loose stools/diarrhea is also a common side effect. The drop-out rate varies in different studies but is around 20–30 %. Much depends on whether the patient is supported to continue during the first weeks of treatment. Patients with tachycardia or palpations should be advised to reduce caffeine intake and, if possible, to increase physical exercise. A small dose of beta blockade may also be useful. For loose stools, loperamide is effective.

Worsening of cardiac insufficiency has been reported, and caution should be used in patients with a previous history of cardiac failure. Coronary insufficiency/angina is not a contraindication [49], and there is no evidence that ANA worsens such symptoms. In the EXELS study, the event rate of atrial fibrillation and other arrhythmias were the same for ANA as for other cytoreductive therapies [15] (Abstract 1344, EHA 2016). Unfortunately, the label advises caution in “heart disease” without discrimination. In clinical practice, cardiac events have been shown to have little impact on discontinuation [50]. Cardiac screening with ECG and/or other measures were shown not to be useful in identifying patients who would develop significant cardiac side effects [51••].

Combination of ANA and aspirin induces an increased risk of haemorrhage [37] (Birgegård et al., ASH 2014, abstract 1846), and the label recommends caution in patients with previous bleedings. There is no concern for leukemogenesis with ANA, and no leukomogenic effect, as shown in large follow-up study [39].

### Dosing

The recommended starting dose is 0.5 mg twice daily, and the rate of increase 0.5 mg per day/week. If the recommended starting dose or increase rate is exceeded, there will be more side effects and in fact a lower efficacy [52••]. This shows that the recommendations in the SPC (label) are well grounded, which also is my own experience. The maintenance dose has been reported as around 2 mg daily in several studies [36, 44, 53]. However, in studies of clinical practice the maintenance dose is lower, 1.5 mg daily [54, 55, 56••].

Switching from ANA to other therapy can either be done gradually, by adding a standard dose of the new drug and gradually removing ANA (for instance with 1 tablet/day/week) as control of platelet levels is achieved. If more rapid cessation is necessary due to severe side effects, ANA can be stopped immediately and, in this situation, HC should be started at the same time. If the patient has a previously known intolerance or lack of response to HC, IFN may be used, but one should be aware that the effect of IFN is slower than that of ANA and especially HC.

### Interferon in ET

The three main treatment alternatives are ANA, HC and interferon (IFN). Since the randomized ANA studies have used HC as a comparator, this drug has been commented on above. Interferon-α (IFN) suppresses growth of multipotent haematopoietic progenitor cells. IFN treatment is well documented and safe in ET [57–60] and there is no theoretical concern or clinical evidence that it is leukemogenic or teratogenic. Pegylated IFN given sc weekly or even every second week has been shown to have equal efficacy as conventional IFN given three times weekly. The efficacy of platelet lowering is similar to that of ANA or HC, but the effect comes slower than for both. Fatigue and mood changes are the most common side effects, causing cessation of therapy in 20–25 % of patients. Myalgia and activation of present or latent autoimmune disease including hypo- and hyperthyreosis are not uncommon. Liver enzymes and thyroid hormones should be monitored. IFN is still not registered for use in ET due to the lack of interest from the IFN-producing companies to make studies in this disease. There is no published randomized study including IFN, but an ongoing with HC as a comparator.

In the Nordic MPN treatment recommendations, pegylated IFN or ANA is first-line therapy in high-risk ET in patients <60 years of age. This is possible because physicians are free to use IFN without limitation based on its registration for other disease, and IFN is reimbursed. Unfortunately, there are formal obstacles for its use in many other countries. In some countries, only the non-pegylated from can be used, and IFN is often not reimbursed.

If cytoreductive treatment is needed during pregnancy, IFN is the only safe choice.

### Treatment Pattern

Anagrelide is registered as second-line therapy in Europe (first line in USA, Japan and some other countries) and is mainly used as such, when there is intolerance or unresponsiveness to HC. However, the use of ANA is strongly influenced by patient age; in the European EXELS study, 53 % of patients younger than 40 years had ANA at registration, and 45.8 % of treatment-naïve patients received ANA [56••], indicating that many doctors choose ANA as first-line therapy in young patients, probably due to the concern for possible leukaemogenecity of HC. Table 1 shows the author´s preferred treatment algorithm.

**Table 1:** The author’s treatment algorithm for ET

< 60 years	First line: Anagrelide or interferon
	Second line: Hydroxycarbamide
>60 years	First line: Hydroxycarbamide
	Second line: Anagrelide or interferon
>75-80 years	First line: Hydroxycarbamide
	Second line: Anagrelide
	Third line: Interferon with great caution, busulphan intermittently.

### Cytoreductive Treatment in ET

Cytoreductive therapy should be given to high-risk ET patients (age > 60 or previous thrombosis or platelets >1500 × 10^9^/L. Patients classified as low risk who have other additional risk factors like cardiovascular risk, especially in combination with JAK2V617F mutation, may also be considered for cytoreduction. The role of leucocytosis is still unclear—it is not included in the IPSET-thrombosis risk score model, even though many doctors use it as an additional risk criterion.

Busulphan is useful in selected patients but has leukemogenic potential, especially if used after or together with other chemotherapeutic drugs. Radioactive phosphorus cannot be recommended due to leukemogenicity and short response duration.

### Treament Goal in ET

The treatment goal in ET is to reduce thrombosis, haemorrhage and symptoms. Since symptom reduction and choice of therapy has not been well studied, the efficacy of treatment is focused on avoidance of thrombosis and haemorrhage through platelet reduction. Normalization of platelet counts is the generally accepted treatment goal, but side effects of given drugs are sometimes dose-limiting and there is a trade-off between side effect tolerance and fulfilment of platelet reduction goals. The balance is sometimes not easily found, especially in the light of recent studies showing a correlation between platelet levels during follow-up and thrombosis [16, 17•], but it must be remembered that the complication rate during cytoreductive therapy is as low as 2–3 %/100 patient years and that the correlation between moderately increased platelets and thrombosis cannot be very strong, considering the failure of many attempts to show it.

### Combination Therapy

In patients with reduced efficacy of given treatment, sometimes because of dose-restricting side effects, combination therapy may be of use. A lower dose of two drugs reduces side effects and gives a high response rate [55, 61]. In my opinion, this should be the preferred third-line therapy in ET. Studies on combination therapy have focused on the combination of HC and ANA, but in clinical practice, also combinations with interferon (IFN) are used, although the latter is not registered for these diseases.

### Anagrelide Use in Polycythemia Vera (PV) and Myelofibrosis (MF)

The efficacy of ANA for platelet lowering is similar to that of ET in PV and MF [35, 36, 44]. In PV and MF, cytoreduction is usually given in order to achieve a general effect rather than just a platelet-lowering. In a limited number of patients, there may be a need to use the specific platelet-reducer ANA. There are no generally accepted recommendations for platelet reduction in PV and MF, but many doctors use the same treatment goal as in ET, normalization of platelet counts in high-risk patients, whereas some doctors disregard platelet numbers unless the level is high enough to induce a risk of bleeding (>1500 × 10^9^/L). In clinical practice, addition of ANA to HC or IFN if platelet treatment goal is not achieved is sometimes used.

## Conclusion

Anagrelide is an important alternative for the treatment of thrombocytosis in MPN, especially in ET. The optimal patient group for ANA treatment is true WHO-defined ET, but for second-line therapy, it is also useful in early PMF, PV and MF. The platelet-lowering and the thrombosis-reducing efficacy is similar to that of HC, although with minor differences in arterial (less effective) and venous (more effective) prophylactic efficacy. Side effects are common during the first weeks of therapy but usually subside to grades 1–2 or disappear.
